# Report of the 2018 annual meeting of the Asia Pacific Malaria Elimination Network Vector Control Working Group: harnessing skills and knowledge for malaria elimination across the Asia Pacific

**DOI:** 10.1186/s13071-021-04778-3

**Published:** 2021-05-29

**Authors:** Latifeh Dahmash, Allison Tatarsky, Fe Esperanza Espino, Theeraphap Chareonviriyaphap, Michael B. Macdonald, Jettsumon Sattabongkot Prachumsri, Pradeep Srivastava, Christina Rundi, Jeffrey Hii

**Affiliations:** 1 Malaria Consortium Asia, Faculty of Tropical Medicine, Mahidol University, Bangkok, Thailand; 2 College of Public Health, Medical and Veterinary Sciences, James Cook University, Queensland, Australia; 3 Malaria Elimination Initiative, Global Health Group, University of California, San Francisco, CA USA; 4 Department of Health, Research Institute for Tropical Medicine, Manila, Philippines; 5 Kasetsart University, Bangkok, Thailand; 6 Sc.D. Consultant, Catonsville, MD USA; 7 Mahidol Vivax Research Unit, Faculty of Tropical Medicine, Mahidol University, Bangkok, Thailand; 8 National Vector Borne Disease Control Programme, Delhi, India; 9 Sabah State Health Department, Ministry of Health, Sabah, Malaysia

## Abstract

The 2018 Asia Pacific Malaria Elimination Network’s Vector Control Working Group (APMEN VCWG) annual meeting took place 3–5 September 2018 in Bangkok, Thailand. It was designed to be a forum for entomology and public health specialists from APMEN country programmes (over 90 participants from 30 countries) to discuss current progress and challenges related to planning, implementing, and sustaining effective vector control (VC) strategies for malaria elimination across the region, and to suggest practical and applicable solutions to these moving forward. The meeting was organised as a joint collaboration between the VCWG host institution—Faculty of Tropical Medicine, Mahidol University, Thailand—and leading partner institutions within the VCWG: Malaria Consortium and the Malaria Elimination Initiative at the University of California, San Francisco, Global Health Group (UCSF Global Health Group), under the leadership of the APMEN Director and VCWG Co-Chairs from ministries of health in Malaysia and India. This report provides an introduction to the role and nature of the VCWG, highlights key themes and topics presented and discussed at the meeting, and outlines the future objectives and focal areas for the VCWG and APMEN at large.

## Background: the role and significance of the Asia Pacific Malaria Elimination Network (APMEN) in the Asia Pacific

### APMEN

Established in 2009, APMEN is a collaboration of 21 country partner National Malaria Control Programmes (NMCP) within the Asia Pacific (AP) committed to eliminating malaria from within their borders by 2030. The Network operates through the provision of technical and programmatic guidance working groups, comprised of national malaria programme representatives and academic and research partners from across the globe, operating under the technical supervision of a secretariat group currently based in Singapore. The working groups are centred around three thematic areas of malaria control and management: vector control (VC), surveillance and response, and control of *Plasmodium vivax* (particularly prevalent across the region). The evolution of APMEN over the last decade (initially composed of nine countries) has enabled country partners to access and build the technical knowledge, tools, and in-country expertise required to advance both individual country and regional malaria elimination goals. This expansion has also seen APMEN form stronger collaborative ties with its policy and advocacy sister organisation, APLMA—the Asia Pacific Leaders Malaria Alliance—with the aim to build a more integrated response to malaria elimination. The strength and leverage of both organisations, as independent bodies and in alliance, has positioned them as critical parts of an inter-country regional platform committed to promoting malaria elimination: APMEN focusing on building, developing, and sharing technical guidance and learning for elimination, and APLMA working closely with political and leadership bodies to communicate and integrate this evidence into focused strategy responses with government bodies across the region.

### The history and role of the Vector Control Working Group (VCWG)

The VCWG was established at the inaugural meeting of APMEN in 2010, consisting of voluntary representatives from APMEN country partners: NMCPs, research institutions, World Health Organization (WHO), and the APMEN secretariat (then based in Brisbane, Australia). The initial mandate was focused upon addressing the dual challenges of insecticide resistance and absence of local vector surveillance across the region, primarily through the provision of technical support to operational research activities.

Over the past decade, and following extensive and ongoing consultation with country partners, the VCWG has expanded its focus to provide support to additional core VC and entomological operations, including operational approaches to insecticide resistance monitoring, species identification methods, larval source management practices, local entomological surveillance strategies, and identifying and trialling new outdoor VC and surveillance tools. Activities developed and supported under each of these include conducting local and cross-country technical trainings, facilitating individual and group fellowship exchange opportunities, and providing hands-on technical support for operational research and entomological monitoring and evaluation within NMCPs, at the local and national level. More recently, technical and programme support has also been provided through the VCWG-led Online Resource Exchange Network for Entomology—ORENE: an online community of practice platform dedicated to sharing technical guidelines and standard operating procedures (SOPs), and providing online training and opportunities for skills development, across entomology and VC.

To strengthen and harmonise existing partner efforts across the region, the VCWG seeks to actively engage, and continues to work closely with, national programmes and implementing partners in both the public and private sectors, including WHO, the Roll Back Malaria Vector Control Working Group (RBM VCWG), and the Mekong Outdoor Malaria Transmission Network (MOMTN). With the evolving needs of Network country partners, and subsequently the role of the VCWG, these core focus areas have been further refined to address and meet current and emerging challenges to regional malaria elimination.

To showcase and reflect on developments and remaining challenges from across the region, the VCWG annual meeting brings together entomology and VC expertise from across NMCPs, academia, the private sectors, non-governmental organisations (NGOs), and civil society organisations. The sessions included, and topics discussed, provide a unique opportunity for cross-sectoral, cross-country learning and to mutually identify, define, and anticipate the current and future priorities for entomological surveillance and VC strategies across the AP, and beyond, as countries transition from malaria control to elimination, as well as develop and share strategies for the prevention of reintroduction of malaria in those contexts where elimination targets have been met. Highlights from the 3–5 September 2018, VCWG annual meeting are described in this report.

## Overview of entomological surveillance and VC in the Asia Pacific in 2018: at a crossroads?

Dr Tessa Knox (WHO) opened the first session of the 2018 APMEN VCWG meeting with a reflection on recent progress of WHO’s technical assistance for entomology in the region. WHO has supported the development of regional action plans, country-led VC needs assessments, and the initiation of an independent needs assessment specific to the Greater Mekong Sub-region (GMS) to identify gaps and challenges to VC coordination and implementation. Special attention was drawn to revised entomological surveillance and monitoring guidelines within the 2018 WHO manual for malaria surveillance, monitoring, and evaluation, emphasising a need for a tailored approach to entomological surveillance, taking into account both technical and programmatic aspects of VC in the context of a ‘malaria elimination continuum’, and new ways of classifying foci in elimination settings. With evidence of insecticide resistance in many areas across the region, Dr Knox also emphasised the need for targeted insecticide resistance monitoring (IRM) strategies that are guided by local situational analyses. WHO is currently tracking resistance trends and gaps in surveillance monitoring for IRM globally, with data showing a notable increase in insecticide resistance and intensity, mainly in the African region and in dominant vector species. Dr Knox also highlighted how this data is being utilised within the Malaria Threats Map initiative—a newly developed tool aimed at tracking biological challenges to malaria control and elimination—as well as for the development and pilot of modules for entomology and VC within the District Health Information Software (DHIS2) increasingly used by countries for surveillance.

Following this, Dr Sean Hewitt (WHO consultant) presented recent findings from a recent WHO review regarding current status, capacity, and knowledge gaps in malaria entomology and VC across countries within the GMS. Results indicated that limited entomological capacity within programmes, poorly managed monitoring and evaluation (M&E) activities, and uneven coverage and suboptimal quality of VC interventions is stalling and endangering control and elimination programmes. Dr Hewitt also highlighted that VC coverage is very low amongst forest-goers, particularly worrying given that the majority of malaria transmission across this region is restricted to densely forested areas frequented by mobile and migrant populations (MMP), such as forest-goers. Also highlighted was low knowledge regarding the entomological and epidemiological drivers of residual malaria transmission (RMT) in these high-risk areas. Whilst new prevention tools to limit RMT in these persistent foci are currently under evaluation (ranging from insecticide-treated clothing (ITC) and airborne repellents, to cattle and human endectocides), attention should be paid to the fact that the current core interventions for malaria VC are invariably effective in interrupting transmission of malaria in specific settings, if supported by strong implementation and management strategies.

Complementary to this research, results were then presented from a recent survey evaluating the current status of vector monitoring across the AP from Dr Tom Burkot (James Cook University, Australia). Whilst there is evidence for granularity of data in some settings across the AP (e.g. data of vector distribution at a village level), almost a third of countries do not collect information regarding vector distribution required to characterise and monitor receptivity status. Data to measure the relative density of vector species is not effectively carried out to determine the annual seasonality of transmission to inform optimal timing of interventions, and data to determine adult resistance mechanisms is rarely collected. Data for entomological indicators used to determine risk for malaria transmission, such as adult biting time and indoor and outdoor biting rate, also remains low. The major challenge noted for conducting efficient vector surveillance activities was inadequate programme capacity (notably human resources), as well as few opportunities for training and skills development. Operational research for the effectiveness and use of alternative surveillance techniques needs to be prioritised to counter the disadvantages of human landing catch (HLC) sampling techniques. A lack of priority given to entomological activities in country malaria strategic plans was also highlighted: technical and programmatic guidelines to facilitate good practice are often not available, especially at the district level. As the arsenal of VC tools begins to expand in the AP, so too the demand and investment in comprehensive surveillance data needs to increase to ensure that selection and deployment of VC tools is both driven and informed by comprehensive, quality data.

## Identifying, monitoring, and responding to insecticide resistance

On the subject of insecticide resistance (IR), Dr Tessa Knox provided an overview of the status for insecticide resistance monitoring (IRM) across the AP, highlighting the role of WHO’s digital data collection and management system to support IRM (through DHIS2 or other information systems), at both the country and global level. Recent analysis of collection and use of IR data for the AP overall highlighted a mixed picture. Whilst many countries within the South Asia region have shown steady progress, evidence suggests little to no data reported for those in the Western Pacific in the last 3 years (potentially due to under-reporting or less investment in IR surveillance in some settings). The majority of the data is focused on discriminating concentration bioassay alone, with little molecular or biological bioassay information reported, and very little data on resistance mechanisms. Importantly, the data showed that there is confirmed resistance to pyrethroid and dichlorodiphenyltrichloroethane (DDT) in most APMEN countries, with wide variation across different settings, and largely driven by India. WHO is currently working with countries in the region to strengthen IR data monitoring and management plans, with focus on supporting routine data collection, reporting, and evaluation to better assess the impact of IR on current VC interventions. This platform will support national authorities to adopt rational and target specific use of insecticides for effective VC.

Following this, Dr Theeraphap Chareonviriyaphap (Kasetsart University (KU), Bangkok) provided an overview of methods for mosquito sibling species identification and behaviour monitoring of outdoor vectors in Thailand [1]. Reflecting on the steady progress Thailand has made in the last decade in reducing malaria incidence and burden of *P. falciparum* cases, Dr Chareonviriyaphap highlighted the need to (a) use more sensitive species identification methods to capture this variability, and (b) understand distinct behavioural characteristics that are key to continued malaria transmission in specific foci. It was emphasised that a combination of morphological and molecular techniques should be encouraged to facilitate correct species identification, as well as to identify specific insecticide resistance mechanisms. KU has also developed geographical maps across Thailand and the wider GMS region, combining data on both vector locations and related behavioural characteristics to help monitor and track species density and composition. Strengthening knowledge and technical skills to identify and monitor vector resistance to insecticide should be a priority at both the national and local level to support evidence-based insecticide use for vector-borne disease control programmes.

Dr Rathchadawn Ngoen-Klan, also from KU, presented her research focused on developing a comprehensive IR monitoring database for Thailand for both *Anopheles* and *Aedes* vector species. Using geographic information system (GIS) technology, the team have mapped both retrospective and current data for IR as well as entomological and epidemiological data to more precisely identify, track, and predict IR patterns, highlight at-risk population groups in these locations, and better inform relevant VC strategies. To date, the tool has helped to identify and track changing patterns of IR in *An. minimus* species across Northern Thailand, identify specific sibling species showing higher levels of IR to different insecticides, and identify different behavioural patterns for insecticide avoidance amongst *An. dirus*, *An. maculatus*, and *An. minimus* species complexes across sites in Thailand.

The session concluded with a presentation of the results of a recent novel study investigating the efficacy of transfluthrin (an alternative, fast-acting pyrethroid with low persistency)-treated polyethylene terephthalate (TFT-PET) sheets against *An. minimus* s.l. in Thailand: a dominant outdoor biting mosquito. Results suggested that TFT-PET provided relatively high (≈ 73% landing inhibition) and low (≈ 30% landing inhibition) protection in semi-open and open field conditions, respectively. It was suggested that the use of TFT-PET could serve as a complementary tool to existing VC tools, and further research is needed regarding public understanding and acceptance of use of transfluthrin.

## Understanding residual transmission of malaria

Dr Knox addressed the topic of residual malaria transmission (RMT), reiterating that both a uniform definition and approach to addressing this challenge to malaria control remain weak. WHO’s RMT guidance document includes best practice approaches to define and quantify the drivers and magnitude of RMT at local level, as well as understand the limitations of current VC tools in specific regions [2]. Importantly, recent results of a WHO-led assessment examining the extent and causes of RMT across four regions (including countries in the GMS and West Pacific Regional Office–WPRO–regions) highlighted that understanding RMT requires a holistic analysis of the gaps and challenges experienced within any malaria control programme, from VC and case management coverage/access, and vector composition, to compliance and use of tools. An isolated focus on RMT alone will only reveal one part of a much more integrated transmission cycle.

Dr Jeffrey Hii (Malaria Consortium/APMEN VCWG) then presented results from a recent mixed-methods WHO-funded study investigating the magnitude of RMT and factors contributing to low, but sustained, malaria transmission in rural communities on the Thai-Myanmar border, and in central Viet Nam [3, 4]. Utilising Global Positioning System (GPS)-enabled tracking devices in three ecological sites frequented by local farmers and community members who slept overnight in villages or hamlets, farm huts, and forest plots, the researchers identified vulnerability hotspots of persistent malaria transmission which are far away from village homes. Results suggested that primary drivers of RMT here were higher mosquito abundance in forested areas where long-lasting insecticide-treated nets (LLINs) were used less frequently or could not be used, feeding preferences of *Anopheles* vectors taking them away from contact with LLINs and indoor residual spraying (IRS), and high population movement across the border and into forested areas. Transmission in these sites presents a formidable challenge to elimination, not only because VC measures are harder to implement and monitor, but also because febrile and sick people have less or no access to nearby health services, putting them at greater risk of prolonged infection. Recommendations included exploration of personal protection methods for use in these areas that require minimal behaviour change and communication strategies. Importantly, the study supported the need for a more holistic definition for RMT, as net use is often overestimated and does not take into account the movements and behaviour of people outside of the households, and lower LLIN access/use in farm huts/forest areas.

## Tools for entomology and vector surveillance

Following the consensus for simplified operational tools from partners across the AP, Dr Neil Lobo from the Global Health Group at the University of California, San Francisco (UCSF) discussed a new entomological surveillance planning tool (ESPT), currently being trialled in both African and Asian settings. Particular strengths of the tool are (a) an emphasis on a set of minimum essential entomological indicators to facilitate a more manageable and tailored approach to programme surveillance efforts and (b) an emphasis on the practical integration of entomological and epidemiological information to guide a more comprehensive monitoring and response processes. The tool provides users with a step-by-step, iterative approach to shape their surveillance strategy, and also highlights effective sampling approaches and designs relevant to best capture different vector species.

Advocating the strength of a unified approach to entomological surveillance, Ms Perada Wilson of the Malaysian Ministry of Health (MOH) presented the national MyFoci entomology database system: a MOH-led entomological surveillance platform developed to assist with accurate foci classification to characterise and stratify malaria risk areas, and to curb re-introduction of malaria in areas of cleared foci. Using short preliminary ecological assessments coupled with entomological surveys and decentralised surveillance, MOH developed a graded receptivity and vulnerability matrix to stratify localities according to malaria risk level (cleared, active, and residual non-active foci). Based on this combined receptivity-vulnerability data, the MOH and local response teams have better customised VC response strategies within each of these areas according to their ecological and demographic profiles.

A novel sampling method for exophilic mosquitoes was jointly presented by Dr Neil Lobo and Dr Frances Hawkes of UCSF and Greenwich University, respectively. Reiterated was the need to tailor mosquito sampling methods/tools that take into account diversity in vector behaviour across different localities, as well as the difficulties in effective sampling of outdoor biting mosquitoes. A new tool—the host decoy trap (HDT)—which utilises a model of host-associated stimuli, showed higher trapping efficacy of HDT in capturing *An gambiae* s.l., *Mansonia* spp., and *Culex* spp. vectors than HLC throughout all season scenarios in a pilot study in Burkina Faso [5]. A multi-country trial (supported by University of Greenwich, the UK Medical Research Council, and Biogents) in sub-Saharan Africa and Asia is currently evaluating a standardised HDT prototype tool to determine its efficacy relative to HLC. Preliminary results across the sites highlighted important behavioural parameters to consider for use, as well as the importance of accurate molecular identification to determine efficacy. The project is now seeking to refine and trial the HDT tool across other sites in the AP to build the evidence base to further determine its efficacy for use in different settings/seasons.

Dr Tom Burkot of James Cook University, Australia, closed the session with a presentation of the barrier fence mosquito monitoring tool: an inexpensive, neutral, non-attractant trap that intercepts the natural flight pattern of all mosquitoes in a given area for capture of blood fed and host-seeking female mosquitoes [6]. Various ‘shade cloth’ materials made of polyvinyl chloride (PVC), coated polyester, polyethylene mesh, or cotton netting were assessed. Trial results from Indonesia indicated that netting colour, placement, and frequency of data collection had an effect on the number and types of mosquitoes captured. Barrier fence monitoring has been highly used across sites in the AP and Latin America, with positive success rates observed [7], and the evidence for efficacy being built further through current trials continuing across both Asia and sub-Saharan Africa.

## Evidence and solutions to address interactions between human and vector behaviour on malaria transmission

Continuing the discussion regarding evidence to better understand the primary drivers of outdoor malaria transmission for VC in the AP, Dr Adisak Bhumiratana (Thammasat University, Thailand) presented research findings looking into the effect of IRS using bifenthrin for *Anopheles* vectors in sites across East and Southern Thailand. Importantly, results showed that IRS using bifenthrin has a positive impact in reducing species composition and abundance—particularly indoor density of primary vectors such as *An. dirus*, *An. minimus*, and *An. Maculatus*—as well as changes in feeding behaviours of *Anopheles* vectors, likely to select for outdoor biting (more detail of the study and results can be found on the APMEN website).

Dr Amelie Vantaux (Institut Pasteur, Cambodia) highlighted results of field studies investigating mosquito activity rhythms and host preference in forested areas of eastern Cambodia, where risk of malaria transmission has been shown to be highest due to low use of LLINs and inadequate housing conditions. Findings showed that almost 70% of mosquitoes collected through human-baited traps were in the forest, mostly during night-time hours, with significantly higher numbers during the rainy season. Just over 4% of all mosquitoes sampled were *Plasmodium*-positive (mainly *P. vivax*), with a higher proportion occurring during night-time hours from human-baited traps. Importantly, the data revealed zoophilic behaviour/generalist host preference (particularly for *An. dirus*—a dominant vector in this region), indicating that infectious mosquitoes may be sustained by animal hosts when humans are not (yet) present.

Focusing on high-risk occupational groups, Dr Masatoshi Nakumara (Japan International Cooperation Agency- JICA- Myanmar) presented data on the relationship between populations working in ‘slash-and-burn’ agricultural areas in Myanmar (where malaria positivity rates remain high despite relatively good access to malaria health services) and seasonal changes of anophelines. Research data revealed that slash-and-burn cultivation in fields gradually provide favourable breeding conditions for *Anopheles* mosquitoes. Importantly, the data highlighted that highly receptive areas for malaria transmission are being created and moving year by year, in line with changing agro-forestry industry practices. Collaboration with local government departments working in the forest is essential to better target VC and case management activities due to restrictions placed on many local NGO partners in Myanmar, and to build a coordinated elimination response amongst state and non-state organisations. An understanding of local ecological conditions, including entomological surveillance, has therefore been crucial to developing data-driven, locally appropriate malaria control responses.

An overview of vector bionomics for primary malaria vectors in the high-risk border provinces of Northern Thailand (*An. dirus* and *An. minimus*) was presented by Dr Wannapa Suwonkerd (Assistant Director of Regional Office of Disease Prevention and Control, Thailand). Findings show that indoor/outdoor biting behaviour in these environments is species- and location-specific, suggesting that conventional VC tools may not always be appropriate, and alternatives should be sought and encouraged. Promising results for plant-based substitutes (*Andrographis paniculate*) have recently been shown in controlling *Aedes aegypti*, with the hope that the active ingredient can also be an effective tool against *Anopheles* vectors in the future. It was also reiterated that primary challenges faced by the MOH here is the lack of skilled entomological staff present in these areas, and lack of research/data available documenting human behaviours and risk for malaria transmission.

Operational research findings from a public–private partner strategy for the distribution of insecticide-treated hammock nets (ITHN) in Vietnam was presented by Dr Joselyn Neukom (Population Services International, PSI). The collaboration between PSI and the Vietnam MOH seeks to better engage with and work with private providers to cater to forest-going population groups in high-risk malaria areas. Within this alliance, qualitative research on user preference for ITHN compared with two different types of nets—manufactured by TANA Netting and Tianjin Yorkool—revealed that, overall, TANA nets are the preferred choice by forest-goers, primarily due to comfortable material, ease of use, extra storage compartments, and perception of protective effects. The limitation is the high cost of TANA canopy nets. Based on this feedback, PSI and the MOH are now working with private outlets and worksites already serving/employing forest sleepers to distribute/promote TANA ITHN for free/at a subsidised price in locations with less access to commercial markets. Following this, PSI plan to conduct a 12-month post-project study to assess improvements in awareness, access, and use of ITHN amongst forest-goers in the same areas to inform the next stage of the research.

Research findings investigating the efficacy of using ivermectin (IVER)—a common drug used to treat filarial parasites of humans and animals that also affects mosquitoes if ingested in a blood meal—to suppress outdoor malaria transmission were presented by Dr Kevin Kobylinski of the Armed Forces Research Institute of Medical Sciences (AFRIMS), Thailand. Mass drug administration (MDA) with IVER has long been used across the African continent for the prevention and treatment filarial parasites, and has been used against a wide range of *Anopheles* vectors globally. In collaboration with partners, AFRIMS have conducted in vitro studies of mosquito survivorship when ingesting IVER alone and when combined with other anti-malarial drugs, with results showing significant decrease in mosquito survival (both *An. dirus* and *An. minimus*) in the GMS [8], as well as IVER MDA field studies showing a similar killing effect for mosquitoes in West African settings. An AFRIMS/Mahidol University-led MDA programme is also currently underway to assess the efficacy of IVER to suppress outdoor malaria transmission across rubber plantation sites in southern Thailand, seeking to measure and evaluate both entomological and epidemiological outcomes.

Insights into anthropological observations of population mobility and malaria risk were presented by Dr Koen Peeters of the Institute of Tropical Medicine (ITM), Antwerp, with an emphasis on the limitations of applying a ‘one-size-fits-all’ malaria elimination model for mobile and migrant populations (MMP), and a need to recognise the diverse behaviour and vulnerabilities of sub-groups within this wider population. Research findings have shown that MMPs are often not officially registered by the state in which they work, and often live and not only work in forest settings, resulting in them being missed/not targeted to receive state-based interventions. Mutual distrust between mobile groups and local inhabitants/country officials can often lead to MMP avoidance of NMCP-managed prevention and treatment services and encourage the use of medicines from unregulated, informal private outlets. A need to encourage and conduct qualitative research alongside quantitative data collection by state and non-state actors is recommended to build and communicate a holistic understanding of MMP needs, and develop more tailored prevention and treatment strategies.

## Methods to accelerate access to VC commodities and technologies

Seeking to better scale up access to VC products, Ms Allison Tatarsky (UCSF) introduced the Standardized Technology Access Mapping System, or STAMPS—a tracking and accountability platform to document and map access to new VC tools for the AP. Whilst a rich pipeline for innovative VC tools does exist, the evidence for these remains largely unrecognised amongst NMCPs and wider stakeholders, and their development and access often restricted by regulatory/policy barriers to market entry. The STAMPS initiative therefore aims to bring greater visibility to this pipeline for relevant bodies by (a) tracking and exposing research and development of new VC tools, (b) highlighting products on the global market that can be introduced into national/regional markets, and (c) improving access to information to improve field evaluation of new tools and expand the evidence base. STAMPS will also explore modelling of potential pathways to markets for various VC tools; consolidate regional groups for policy makers, donor agencies, and industry to articulate disparities and highlight opportunities for engagement and market entry of new technologies; and track countries’ adoption of policy changes as new information regarding new VC tools becomes available.

This was followed by a presentation of Dr Marion Law (WHO) with a reflection on recent progress to accelerate access to new and existing VC products through the WHO pre-qualification (PQ) team (previously WHOPES). Following the second WHO PQ-led Assessors meeting in Tanzania in 2018, some challenges/barriers for registration for stakeholders noted in this region were inconsistencies in label quality, out-of-date evaluation data, and partial product evaluations (particularly for entomological efficacy of pyrethroids + piperonyl butoxide LLINs) against specific IR mosquito populations. The PQ team plan to work on a strategy to better define requirements for PQ vector control product (VCP) labels, support re-evaluation of active ingredients within existing products, and implement a plan for prioritised product reviews. Plans for 2019/20 include identifying and engaging representatives from countries who already evaluate VCPs; documenting and understanding the different approaches and focus in the different jurisdictions/regions to develop targeted strategy plans; and investigating opportunities to harmonise product registration processes with relevant stakeholders and partners.

To conclude this session, Dr Jason Richardson of the Innovative Vector Control Consortium (IVCC) discussed the role of product development partnerships (PDPs) to facilitate innovation for VC tool development within more challenging market spaces. Funded by the Australian Department for Foreign Trade (DFAT), IVCC is leading a multi-partner 4-year project—‘Developing a VC toolbox to support malaria and other vector-borne disease control in the Indo-Pacific’—which will assess the applicability and feasibility of current VC development pipeline products in the AP with a focus on two primary activities: a technical review of VC gaps and opportunities, and an access review to better understand specific country requirements. The organisation will work with multiple partners across the region to determine the safest, quickest, and most impactful route to get these products onto the market for use.

## Network-to-network information sharing and capacity building

The APMEN VCWG ORENE platform was presented by Dr Michael Macdonald: an online country-led community of practice initiative that will also allow country partners and others across the AP to share experiences with different VC methodologies (for both *Anopheles* and *Aedes* vectors), tools and best practices, as well as reflect on shared challenges and locally adapted solutions. Importantly, the platform will provide a space for career and skills development in entomology and VC, through both online training programmes and access to information regarding practical skills training/fellowship programmes across the AP. The website is currently in development (led by Miss Tiff Dahmash of APMEN VCWG and Dr Macdonald) and is scheduled to be launched in 2019.

A similar initiative was presented by Ms Chelci Squires of the London School of Hygiene & Tropical Medicine (LSHTM). Led by LSHTM, the Global Vector Hub (GVH) for VC and entomology will serve as an open-access, interactive technical resource centre for VC and entomology, with the intention to harmonise and condense existing country/regional information networks into one centrally led platform. Through this integration of resources, the platform aims to build programme capacity to facilitate rapid response to vector-borne disease outbreaks and increase the visibility of evidence and guidance tools for implementing appropriate VC tools. The GVH will host free research tools, guidelines, and training packages to enable researchers, local healthcare providers, and practitioners to access up-to-date resources, as well as develop a real-time interactive disease map, with vector and population information. The platform is currently in development and is set to launch in the coming year.

The final presentation of the session was led by Dr Cecil Hugo of ACTMalaria, providing information regarding the first joint training (APMEN, WHO, ACTMalaria, and the Malaysian MOH) for vector surveillance in the AP, to be held in November 2018 in Kuala Lumpur, Malaysia. The course brings together entomology and VC specialists from across the AP region, and globally, with the aim to develop the capacity of entomologists and VC staff, including technicians involved in malaria control and elimination programmes.

## Conclusions and progress in implementing recommendations from the 2018 VCWG meeting

A final feedback session for all participants was held on the final day of the meeting, providing an opportunity to reflect on each of the topic areas, as well as share ideas and thoughts as to priority areas for the VCWG moving forward. Reflecting on the recommendations and action areas for effective entomology and VC outlined in the WHO Global Technical Strategy for Malaria 2016–2030 and the WHO Global VC Response 2017–2030, as well as country NMCP malaria control strategies, a joint exercise was conducted with meeting participants to identify where and how VC and entomological initiatives were lacking/less effective across different settings, and the potential impact of these deficiencies in reaching country, regional, and global malaria control and elimination targets. Participants then collectively listed specific areas in which the APMEN VWCG could provide additional support to address the technical and knowledge gaps identified, given the extent of expertise offered across the Network, with the intention for these to be further presented and discussed with individual ministries of health and NMCPs (Appendix 1).

Whilst steady progress has been made across the AP toward malaria elimination, there is a need to ensure that financial, technical, and logistical resources are not compromised as countries approach their elimination targets (as has been noted) and that stronger accountability mechanisms are adopted and strengthened. Dr Effie Espino (VCWG Secretariat) reiterated that the VCWG will be aiming to build a stronger regional network in the coming year, as many countries approaching/achieved elimination status have much to share with counterparts in the region regarding best practices, lessons learned, and challenges faced along the route to elimination. A focus on targeted research (e.g. new tools for RMT and outdoor), knowledge and information exchange, and improved programme management will take priority for the VCWG moving forward.

A final address from Dr Christina Rundi (VCWG Co-Chair) drew attention to APMEN’s unique position to drive and facilitate new knowledge and capacity building to address key VC/entomology challenges to malaria elimination in the region, encouraging country partners and stakeholders alike to continue to their participation and support within the Network. The wealth of knowledge now evident in the Network, alongside increasing resources at its disposal, presents a great opportunity to lead the way for VC and entomology for malaria elimination across the region:

‘*We’ve come a long way since we started in 2009: we’ve brought new passengers onboard the elimination train, driven the emphasis for the importance of VC for elimination, and built and refined a more definite strategy to achieve our objectives—all of which we will continue to strengthen together in the coming years. Crucially, we’ve shown that the voice of the entomologist can, and will, be heard in the fight for a malaria-free Asia Pacific.’*

## Data Availability

Not applicable.

## Appendix 1

See Table

**Table 1:** Summary of priority areas and recommendations for VCWG support as identified by key representatives from partner organisations

Group	Summary of entomological priorities	Potential areas for APMEN support in partnership with NMCPs
Group 1: Eliminated/elimination goal 2020 (Sri Lanka, China, Korea, Malaysia, Bhutan)	Technical: Maintain knowledge, skills, and commitment of staff in low-transmission settings. Competing health priorities a significant issue (funding and skill set) Reorient role of national and local entomologists from a largely administrative to more technical role Leverage current entomological capacity for arbovirus surveillance to sustain malaria activities Upskilling cadres at lower end of health system in basic entomological skills Engage communities in appropriate, low-resource surveillance and control strategies Develop checklist of minimal requirements for entomological surveillance at the local and national level	Provide trainings for tailored IR surveillance, morphological (identification and analysis techniques) Facilitate south-south trainings: capitalise on lessons learned from near elimination/elimination country/settings Support training on basic vector bionomics studies Support research collaborations, e.g. for IR mechanics Support regional short courses, both face-to-face and virtual (regional, global) Help to develop techniques to track exported cases and facilitate communication between relevant bodies
Group 2: Elimination goals 2021–27 (Laos, Nepal, Cambodia (pf), Thailand, Vietnam, Myanmar (pf. in 5 regions/states), Bangladesh (pf in 8/13 endemic districts), Pakistan (reduce burden by 75% 2020)		Help to engage private sector for harmonised product registration process Support guidelines regarding efficient product procurement for VC Engage with universities to provide capacity-building/training opportunities for the next generation of entomologists Initiate cross-border meetings for idea/strategy sharing Identify and facilitate laboratory support for entomological activities at a local level Identify and encourage donor sustainability and engagement techniques Create a platform for sharing of technical and programmatic resources and tools Develop evaluation tools for entomological surveillance (resources and guidance material) Help to develop techniques to track exported cases and facilitate communication between relevant bodies Support the development of a reporting system for imported/exported cases across borders Facilitate more focused collaboration with the APMEN Surveillance and Response Working Group (SRWG)
Group 3: Elimination goals 2027–30 (Indonesia, Philippines, Vanuatu, India)	Technical: Develop techniques for accurate characterisation/stratification of different malaria transmission areas More accurately understand the correlation between epidemiological and entomological data to guide strategy development/tool investment (promote data-driven programme reorientation) Align/triangulate data and research related to VC and entomology through multi-sectoral collaborations (e.g. research groups, MOH university groups) Standardising memoranda of understanding (MOUs) for research and other partners involved in malaria control activities to support and empower NMCPs (identifying and communicating clear roles and responsibilities of different parties)	Facilitate and advocate for capacity-building activities (trainings, research fellowships opportunities) Support linkage between vector bionomics (species identification and behavioural traits) and epidemiological data Support communications development to generate and report data beyond the local level Provide focused training on development and implementation of SOPs for field/lab studies
Group 4: High burden (Afghanistan, PNG, Solomon Islands)		

1

## Abbreviations

AP: Asia Pacific
APMEN: Asia Pacific Malaria Elimination Network
APLMA: Asia Pacific Leaders Malaria Alliance Secretariat
GMS: Greater Mekong sub-region
HDT: Human decoy trap
HLC: Human landing catch
ITHN: Insecticide-treated hammock net
IRM: Insecticide resistance monitoring
IR: Insecticide resistance
IRS: Indoor residual spraying
IVER: Ivermectin
KU: Kasetsart University
LLIN: Long-lasting insecticide-treated net
NMCP: National Malaria Control Programme
MOH: Ministry of Health
MMP: Mobile and migrant population(s)
PQ: Pre-qualification
PSI: Population Services International
RMT: Residual malaria transmission
STAMPS: Standardized technology access mapping system
TFT-PET: Transfluthrin-treated polyethylene terephthalate
WHO: World Health Organization
VCWG: Vector Control Working Group
VC: Vector control
UCSF: University of California, San Francisco

## References

[1] Tanachai C, Manguin S, Bangs M, Chareonviriyaphap T (2019). Malaria vectors and species complexes in Thailand: implications for vector control. Trends Parasitol.

[2] World Health Organization. Control of residual malaria parasite transmission: guidance note- September 2014 (2014). https://www.who.int/malaria/publications/atoz/technical-note-control-of-residual-malaria-parasite-transmission-sep14.pdf. Accessed 02 Dec 2020

[3] Edwards H, Chinh V, Duy B, Thanh P, Thang N, Trang D (2019). Characterising residual malaria transmission in forested areas with low coverage of core vector control in central Viet Nam. BMC Parasites Vectors.

[4] Edwards H, Sriwichai P, Kirabittir K, Prachumsri J, Chavez I, Hii J (2019). Transmission risk beyond the village: entomological and human factors contributing to residual malaria transmission in an area approaching malaria elimination on the Thailand-Myanmar border. Malaria J..

[5] Hawkes F, Dabire R, Sawadogo S, Torr S, Gibson G (2017). Exploiting *Anopheles* responses to thermal, odour and visual stimuli to improve surveillance and control of malaria. Sci Rep.

[6] Burkot T (2013). Barrier screens: a method to sample blood-fed and host-seeking exophilic mosquitoes. BMC Malaria J.

[7] Davidson J, Supratman S, Asih P, Syafruddin D, Baskin R (2018). Using barrier screens to characterize mosquito composition, flight activity, and abdominal status in South Lampung, Indonesia. BMC Parasit Vect..

[8] Kobylinski K, Ubalee R, Ponlawat A, Nitatsukprasert C, Phasomkulsosil S (2017). Ivermectin susceptibility and sporontocidal effect in Greater Mekong Subregion *Anopheles*. BMC Malaria J.

